# Molecular signatures for obesity and associated disorders identified through partial least square regression models

**DOI:** 10.1186/s12918-014-0104-4

**Published:** 2014-08-30

**Authors:** Neeraj Sinha, Sachin Sharma, Parul Tripathi, Simarjeet Kaur Negi, Kamiya Tikoo, Dhiraj Kumar, Kanury VS Rao, Samrat Chatterjee

**Affiliations:** 1 Immunology Group, International Centre for Genetic Engineering and Biotechnology, Aruna, Asaf Ali Marg, New Delhi 110067, India; 2 Present address: Drug Discovery Research Centre, Translational Health Science & Technology Institute, Gurgaon 122016, India

**Keywords:** Obesity, Type-II diabetes, Gene signature, Partial least square model, Biological classifications, Hub-proteins

## Abstract

**Background:**

Obesity is now a worldwide epidemic disease and poses a major risk for diet related diseases like type 2 diabetes, cardiovascular disease, stroke and fatty liver among others. In the present study we employed the murine model of diet-induced obesity to determine the early, tissue-specific, gene expression signatures that characterized progression to obesity and type 2 diabetes.

**Results:**

We used the C57BL/6 J mouse which is known as a counterpart for diet-induced human diabetes and obesity model. Our initial experiments involved two groups of mice, one on normal diet (ND) and the other on high-fat and high-sucrose (HFHSD). The later were then further separated into subgroups that either received no additional treatment, or were treated with different doses of the Ayurvedic formulation KAL-1. At different time points (week3, week6, week9, week12, week15 and week18) eight different tissues were isolated from mice being fed on different diet compositions. These tissues were used to extract gene-expression data through microarray experiment. Simultaneously, we also measured different body parameters like body weight, blood Glucose level and cytokines profile (anti-inflammatory & pro-inflammatory) at each time point for all the groups.

Using partial least square discriminant analysis (PLS-DA) method we identified gene-expression signatures that predict physiological parameters like blood glucose levels, body weight and the balance of pro- versus anti-inflammatory cytokines. The resulting models successfully predicted diet-induced changes in body weight and blood glucose levels, although the predictive power for cytokines profiles was relatively poor. In the former two instances, however, we could exploit the models to further extract the early gene-expression signatures that accurately predict the onset of diabetes and obesity. These extracted genes allowed definition of the regulatory network involved in progression of disease.

**Conclusion:**

We identified the early gene-expression signature for the onset of obesity and type 2 diabetes. Further analysis of this data suggests that some of these genes could be used as potential biomarkers for these two disease-states.

## Background

The World Health Organization (WHO) recently published that there are over 500 million adults in the whole world who were found to be clinically obese [1]. Obesity is described as an accumulation of adipose tissue and caused by a combination of environmental factors such as excessive dietary calorie intake, lack of physical activity and genetic susceptibility [2],[3]. In spite obesity has been recognized as a problem for decades, it is still on the rise and gradually gaining an epidemic status swapping malnutrition and infectious diseases [2]. Moreover, obesity may cause other health problems, like development of type 2 diabetes mellitus (T2D), coronary heart disease, certain form of cancer etc. [4]. Among these health problems, T2D showed a major link with obese patients and thus undoubtedly comes up as another intriguing health problem of 21st century. T2D is a syndrome with a diverse phenotype, which is not only marked by hyperglycaemia, but also by dyslipidaemia i.e. elevated triglyceride (TG), and elevated plasma free fatty acid (FFA) levels.

Although pharmaceutical approaches to track the problem of obesity and its related disorders are being aggressively pursued, approaches evaluating treatment with alternative forms of herbal medicine are also of interest. For instance, Shao *et al.*[5] showed that curcumin is effective in the treatment of obesity and diabetes. More recently Tikoo *et. al*. [6] showed that KAL-1, a formulation derived from Ayurveda which a system of Indian Traditional Medicine, was extremely effective at suppressing the development of diet-induced obesity in the mouse model. Importantly, development of other related disorders of type 2 diabetes and systemic inflammation was also prevented.

Diet is one of the widespread environmental determinants for the onset of obesity and T2D. Thus, to study obesity and diabetes, we employed the diet-induced mouse model where mice were fed on a “high calorie diet” consisting of high-fat and high-sucrose (HFHSD). Our initial experiments involved two groups of mice, one on HFHSD and the other on normal diet (ND). HFHSD fed mice were then further separated into subgroups that either received no additional treatment, or were treated with different doses of the Ayurvedic formulation KAL-1. Experimental results, based on the physiological parameters like body weight, blood glucose levels and cytokine profiles, confirmed the earlier findings [6] that KAL-1 prevented mice from both obesity and diabetes. Thus, by comparing between the different groups, our experimental system offered an opportunity to identify disease-specific perturbations in gene expression in different tissues. Further, it was also our intent to employ this data for the subsequent extraction of gene expression signatures that specified progression towards diabetes and/or obesity.

So, a primary aim of the present study is to get a gene/molecular signatures for obesity and diabetes by establishing a relation between the early gene expressions and late body response. These molecular signatures could be efficiently used to evaluate relevant biological formulations in a quick and very efficient rapid assay system based on gene expression profiling.

To obtain the molecular signature we developed a model using partial least square discriminant analysis (PLS-DA) method [7]. This algorithm is useful when multi-collinearity exists among explanatory variables and when the number of explanatory variables is very large compared to the number of observations. PLS-DA analysis reduces the multiple dimensions of data set to a principal component space and regress independent and dependent principal components. In our case, we used this algorithm model to find the relationship between the early gene-expression values (independent variable) and late physiological responses like body weight, blood glucose levels or cytokine profiles (dependent variable). We succeeded in identifying a set of genes whose early expression pattern correlated with the subsequent development of obesity and diabetes. A subsequent interrogation of the resulting tissue-specific gene expression signatures then helped to distinguish those genes, or groups of genes (modules), that may play a significant role in driving disease progression.

## Methods

### Animal experiment

**Figure 1:**
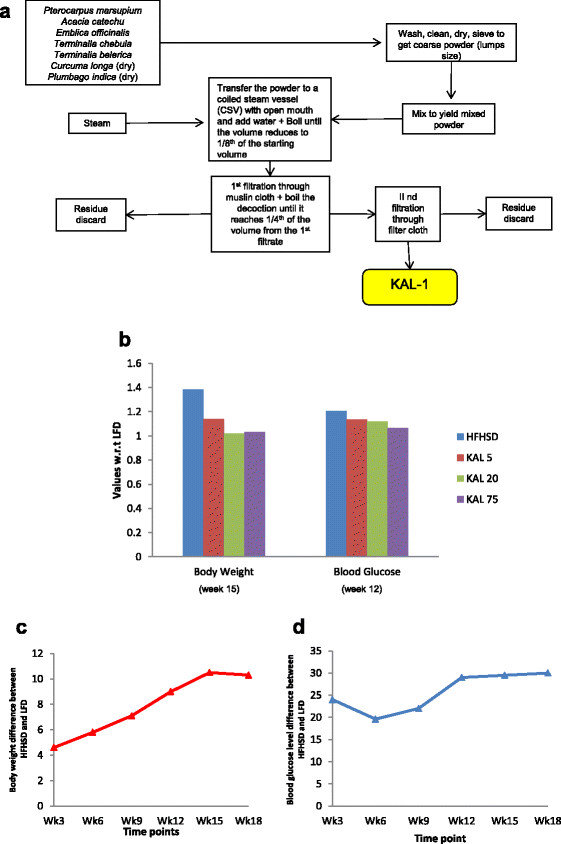
**KAL-1 formulation and its effect on body weight and blood glucose. (a)** Procedure for preparation of KAL-1 kwath. Flow chart showing the step-wise preparation of Ayurvedic formulation termed as KAL-1[6]. This formulation was given to the mice at different concentration (5 μl, 20 μl and 75 μl) along with HFHSD. **(b)**-- **(d)** Body weight and Blood glucose results. **(b)** The difference between the body weights of the HFHSD mice and ND mice is given for each time point. **(c)** The difference between the blood glucose levels of the HFHSD mice and ND mice is given for each time point. **(d)** The average body weight of different group of mice at week 15 and blood glucose of different group of mice at week 12 with respect to the body parameter of ND mice is given.

All animal experiments were performed at BIONEEDS (Laboratory animals & Preclinical services) Bangalore, India, and approved by institutional animal ethics committee (IAEC). BIONEEDS is approved by committee for the purpose of control and supervision of experiments on animals (CPCSEA), Ministry of forests and environments, Government of India. In the present study, we used the C57BL/6 J mouse which is known as a counterpart for diet-induced human diabetes and obesity model since this strain accumulates adipose tissue mass, insulin resistance, hyper-insulinemia, and hyper-lipidemia which is very similar to humans fed on an HFHSD [8]–[10]. The mice were kept at 12:12 hr light: dark photoperiod with ad libitum access to food and water. Weaned mice at the age of 3-4 weeks were divided randomly into five groups- (each group contains 30 animals): first group was fed on ND with 10% of calories coming from fat, the second group was HFHSD fed group with increased sucrose and 60% of calories coming from fat (Research diets Inc.USA), and the third, fourth and fifth group of mice were fed on HFHSD with different doses of an ethano-botanical formulation, KAL-1 and we named them as KAL-5 (5 μg of KAL-1+ HFHSD), KAL-20 (20 μg of KAL-1+ HFHSD) and KAL-75 (75 μg of KAL-1+ HFHSD). The daily weight of this aqueous formulation was 700 mg/ml. A schematic diagram showing the preparation of KAL-1 is given in Figure 1(a).

We generated vast data sets for gene-expression across different tissues from mice being fed on different diet compositions. Tissues were selected on the basis of their anticipated role in obesity, diabetes, or inflammation. The selected tissues were liver, skeletal muscle, brown adipose tissue (BA), white adipose tissue from the epididymal (EA) and subcutaneous (SA) regions, and purified infiltrating macrophages (SVCs) from each of these adipose tissue sites [11]–[13].

The mice were periodically monitored over an eighteen week period. At the interval of every 3 weeks, five animals from each group were sacrificed and different tissues were extracted and frozen in liquid nitrogen prior to RNA extraction. The SVC macrophages and adipocytes tissues were further processed separately using standard protocols- in brief, adipose tissue (BA, EA and SA regions) were isolated, weighed and collagenase solution was added in the concentration of 3 ml/g of the tissue. Further, tissues were homogenized and the tissue solution was kept in shaking water bath at 37°C for 45 minutes. After centrifugation at 3600 rpm for 26 minutes, the pellet was treated with erythrocyte lysis buffer and adipocytes layer was saved for further processing. The treated pellet was centrifuged at 3500 rpm/10 min and the resulting pellet was dissolved in 1x PBS, further, biotin binder beads were added and solution was collected as SVC macrophages. The adipocytes layer obtained was further processed with collagenase and incubated at 37°C for 30 minutes in shaking water bath. After centrifugation at 3600 rpm for 20 min pellet was obtained and was kept as adipocytes. The SVC macrophages and adipocytes for all the three tissues were frozen and sent for RNA extraction.

Isolated tissues were then used to extract gene-expression data for different time points (week3, week6, week9, week12, week15 and week18) for all tissues through microarray experiment. Simultaneously, we also measured different body parameters like body weight, blood Glucose level and cytokines profile (anti-inflammatory & pro-inflammatory) at each time point for all the groups, see Figure 1(b).

### Normalization and noise filtration in micro-array data

The gene-expression data were normalized according to Agilent protocols. Probe level data were summarized into a single expression value for each gene on each array using GCRMA in GeneSpring GX 11 (Agilent Technologies, http://www.chem.agilent.com/). GeneSpring (http://genespring-support.com/files/gs_12_6/GeneSpring-manual.pdf, page 919 and page 920) take care for probable batch effect. Array normalization involved non-linear background reduction, quantile normalization, and summarization by median polishing [14]. Moreover, for each time point we normalize the test data with the control data (ND) to take care of week-wise variation.

In the normalized data we have probe name with Gene Bank accession, gene symbol, name and description. Further for each probe we have three replicate samples and their respective geometric mean with p-value. The gene expressions in the list were given in log_2_-scale ratio between different groups with ND group. A gene is said to be significantly regulated if its expression value is greater than 1 (up-regulation) or less than −1 (down-regulation) i.e., two folds up or down with respect to the control. If the expression value lies between −1 and 1, then the gene is said to be insignificantly perturbed. To remove noise from the data and to maintain consistency we selected only those genes which showed same kind of regulation throughout all the three replicates. Further, in this filtered gene list, we observed that there are some genes present in more than one probe and have different expression values. Therefore, as a final step of gene selection, we select those genes from the duplicate probes that have geometric mean with minimum p-value (2-tailed T-test). This non-redundant (and noise free) set of genes obtained for each time point (i.e., week3, week6, week9, week12, week15 and week18) is called the master list (see Additional file 1 where the master list for week 3 and week 6 is given for Skeletal and Adipose SA for HFHSD, KAL-5, KAL-20 and KAL-75).

### Model formulation

We followed modelling approach that is based on a multivariate statistical method called Partial least square discriminant analysis (PLS-DA) [7]. We used SIMCA P + from Umetrics to do this analysis [15]. The main goal of the PLS-DA model is to predict a set of dependent variable (*Y*) from a set of independent variables (*X*). For the PLS-DA model we first defined an independent and a dependent set of variables.

When the models were run, each of them produced a particular **R2** and **Q2** value. **R2** is the percent of variation of the training set – *X* with PCA and *Y* with PLS – explained by the model. **R2** is a measure of fit, i.e. how well the model fits the data [15]. A large **R2** (close to 1) is a necessary condition for a good model. **Q2** is the percent of variation of the training set – *X* with PCA and *Y* with PLS – predicted by the model according to cross validation. **Q2** indicates how well the model predicts new data. A large **Q2** (**Q2** > 0.5) indicates good predictivity. Poor **Q2** values were generally obtained either when the data are noisy, or if the relationship *X → Y* is poor, or when the model is dominated by a few scattered outliers. In the trained model, the independent variables are ranked depending on a score called variable importance in projection (VIP). The scores are obtained from the graph of observed versus predicted value and was a measure for the predictive ability of the variable with respect to a particular parameter (for details see Additional file 2). Using a cut off value for the VIP score (Additional file 2) we select the top ranked variables and then again built our model with those selected variables.

## Results

### Extraction of the molecular signature for obesity and diabetes

In PLS-DA model, we took gene-expression values as the set of independent variables and dependent variable were the physiological parameters like body weight for the first model, blood glucose levels for the second model, and pro- and anti-inflammatory cytokine profiles for the third and fourth model respectively. The selected pro-inflammatory cytokines were GM-CSF, IFNγ, IL-1a, IL-1b, IL-6, MCP1, TNF-α and IL-12p70 and the anti-inflammatory cytokines were IL-4, IL-10 and IL-13.

In training the model, instead of one week, we considered gene expression values from two different weeks. This increased the range for independent variable. As a result of this increased variability our model could capture wider range of unknown data set for prediction and thus would decrease its chances for failure. We trained all our models with early time point gene-expression values as the independent variable. We chose gene-expression values from week 3 and week 6 (we selected genes common to both the weeks from the master list). In tissues, where this protocol did not work (i.e., where the model training failed), we combined those two weeks (week 3 and week 9) and took the expression profile of the union list as the independent variable. The disadvantage with the latter protocol was that there were some genes present only in one week (in the master list) and so their expression for the other week was kept blank in the model, which made the model weak. Nevertheless, we generated a model with the union list that runs when the intersection protocol fails.

For the dependent variable, as mentioned earlier, we chose different body parameter. The selections of the critical weeks for each body parameter used in different models were decided from the experimental data. The critical week is the time point where the respective body parameter reaches stability i.e., beyond this critical time point the difference between the body parameter of the HFHSD mice and ND mice becomes constant. The critical week for body weight was found to be week 15 and that for blood glucose was week 12 (see Figure 1(c)-(d)). For the cytokines (anti and pro-inflammatory) we observed the critical week to be week 9 (data not shown). However, we were unable to develop a good model using data only from week 9. Therefore, the results of week 9 and week 12 were combined and this approach worked well for both anti- and pro-inflammatory cytokines. Thus for the first model we chose the body weight of week 15 as the dependent variable. For second model, we used blood glucose levels (week 12). For third model we used anti-inflammatory cytokines (week 9 and week 12) and for the fourth model we used pro-inflammatory cytokines (week 9 and week 12).

**Figure 2:**
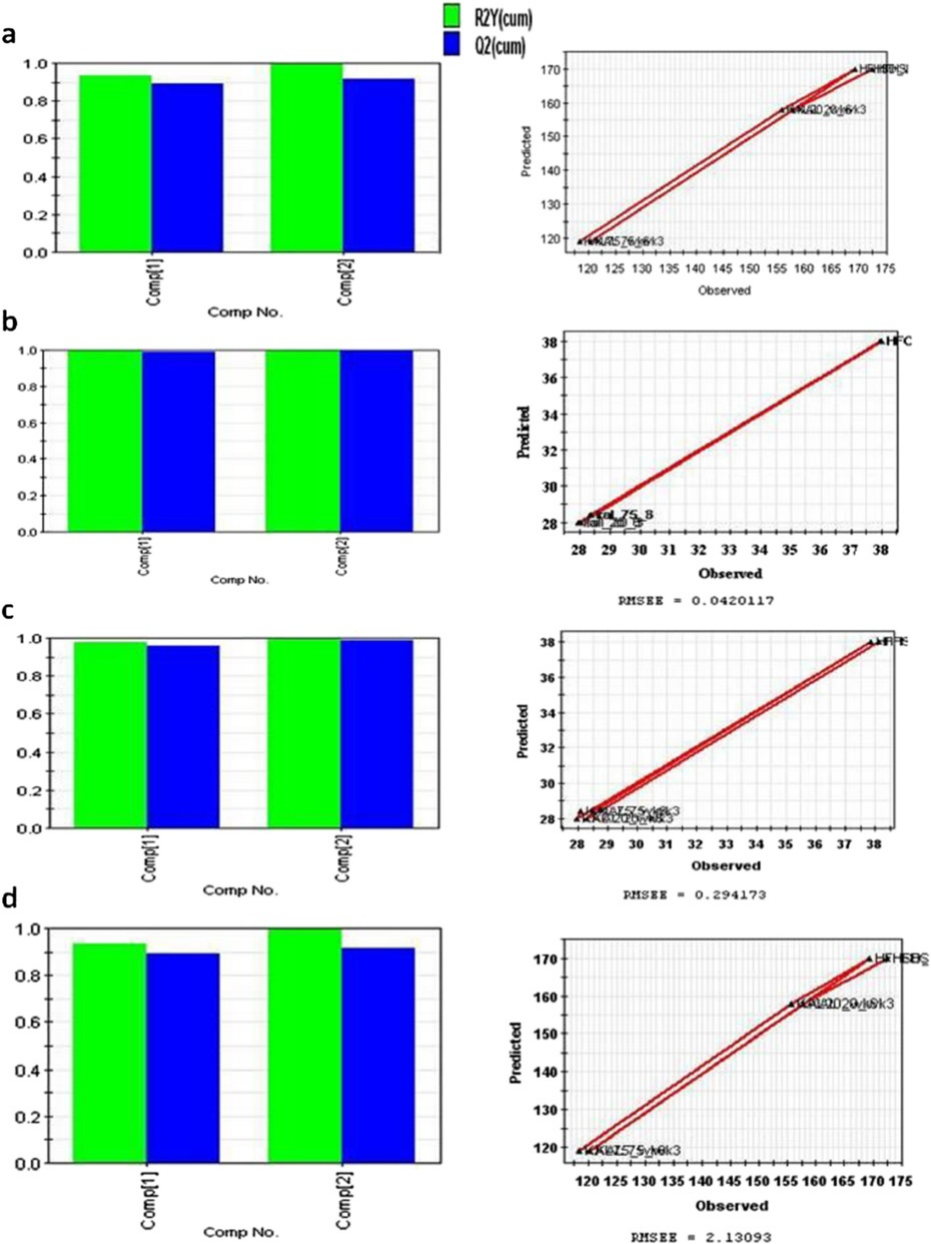
**The figure showing (left side) R2-Q2 value for body weight and blood glucose training model and (right side) observed vs. predicted graph for the same model. (a)** body weight model for skeletal tissue; **(b)** blood glucose model for skeletal tissue; **(c)** body weight model for adipose tissue; **(d)** blood glucose model for adipose tissue.

**Figure 3:**
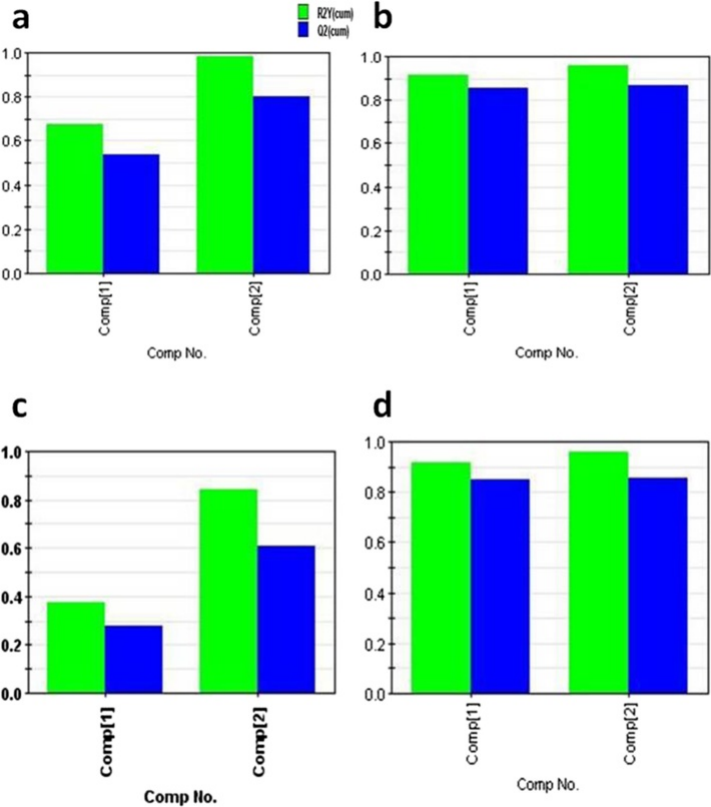
**The figure showing R2-Q2 value for cytokines (anti-inflammatory and pro-inflammatory) training model. (a)** anti-inflammatory cytokine model for skeletal tissue; **(b)** pro-inflammatory cytokine model for skeletal tissue; **(c)** anti-inflammatory cytokine model for adipose SA tissue; **(d)** pro-inflammatory cytokine model for adipose tissue.

The model was constructed for each tissue and the results are summarized in Table 1. It was observed that models satisfactorily worked only for the SA white adipose tissue and skeletal muscle. The **R2**-**Q2** value for Skeletal and adipose tissue is given in Figures 2 and 3. Therefore, we focused on these two tissues and generated eight models (four models for each tissues corresponding to four physiological parameters), the models are given in details in Additional file 3. The models consist of finite number of genes, selected based on the VIP cutoff (see Table 2). Among these two tissues, skeletal muscle yielded better results as it worked for the set of genes that were commonly perturbed at both week 3 and week 6. For the adipose tissue on the other hand, we had to use a combined list of genes from both time points (union set) as a result of which the independent variable data set also included genes that were perturbed at only one of the two time points and the obtained model yield weak predictions. Consequently, we focused only on the skeletal muscle as the most appropriate tissue for deriving the predictive models of interest.

**Table 1:** Table summarizing tissue-wise model result for different body parameters

**Tissues**	**Body weight model**	**Blood glucose model**	**Anti cytokine model**	**Pro cytokine model**
**Adipose BA**	Model worked	Model worked	Model not worked	Model worked
**Adipose EA**	Model not worked	Model not worked	Model worked	Model worked
**Adipose SA**	Model worked	Model worked	Model worked	Model worked
**SVC BA**	Model worked	Model worked	Model not worked	Model worked
**SVC EA**	Model not worked	Model worked	Model worked	Model worked
**SVC SA**	Model not worked	Model not worked	Model not worked	Model not worked
**Skeletal**	Model worked	Model worked	Model worked	Model worked
**Liver**	Model not worked	Model not worked	Model worked	Model worked

**Table 2:** Model summarizations for both skeletal and adipose SA tissue

**Tissue name**	**Model (body parameter)**	**No. of genes**	**Groups used**	**Input week (gene expressions)**	**Output week (physiological result)**
**Skeletal**	Body weight	150	HFHSD, KAL-20, KAL-75	Week 3 and week 6	Week 15
	Blood glucose	168	HFHSD, KAL-20, KAL-75	Week 3 and week 6	Week 12
	Pro-inflammatory cytokines	160	HFHSD, KAL-20, KAL-75	Week 3 and week 6	Week 9 and week 12
	Anti-inflammatory cytokines	182	HFHSD, KAL-20, KAL-75	Week 3 and week 6	Week 9 and week 12
**Adipose SA**	Body weight	147	HFHSD, KAL-20, KAL-75	Week 3 or week 6	Week 15
	Blood glucose	133	HFHSD, KAL-20, KAL-75	Week 3 or week 6	Week 12
	Pro-inflammatory cytokines	144	HFHSD, KAL-20, KAL-75	Week 3 or week 6	Week 9 and week 12
	Anti-inflammatory cytokines	186	HFHSD, KAL-20, KAL-75	Week 3 or week 6	Week 9 and week 12

For model training, we used experimental data consisted of groups from ND, HFHSD, KAL-20 and KAL-75. Thus for model testing we could use unknown data of KAL-5 group. The gene-expressions of KAL-5 for week 3 and 6 were used to predict the body weight for week 15, blood glucose level of week 12 and cytokines (anti-inflammatory & pro-inflammatory) for week 9 and week 12. In the new model, we put the gene-expression of the selected genes under their respective gene names in the *X* matrix (for the independent set) and kept the parameter corresponding to that row empty in the *Y* matrix (for the dependent set). If any gene is not present in the new set due to noise filtration, we kept the spot empty in the *X* matrix. Once the model is run we obtained the **R2** and **Q2** values explaining the goodness and predictive ability of our model. We compared the predicted values with the experimental value and the results are given in Table 3. It was observed that body weight and blood glucose level were better models in terms of consistency and thus can be used to test biological formulations against obesity and diabetes.

**Table 3:** Test results for KAL-5 and comparison of results with the actual experimental results to check the precision of the model in terms of prediction

**Body response parameter**	**Input time (gene expression)**	**Output time (body parameter)**		**Observed**	**Predicted**
**Pro- inflammatory cytokine**	Week 3	Week 9	GMCSF	55	56.1
			IFNγ	71	76.7
			IL1a	103	98.1
			IL1b	54	87.1
			IL6	62	102.6
			MCP1	92	85
			TNFα	92	101.8
			IL12P70	89	102.5
	Week 6	Week 12	GMCSF	60	67.3
			IFNγ	99	89.7
			IL1a	92	128.4
			IL1b	84	117.9
			IL6	97	129
			MCP1	97	95
			TNFα	93	155
			IL12P70	77	124.7
**Anti- inflammatory cytokine**	Week 3	Week 9	IL4	41	52.2
			IL10	63	68.3
			IL13	66	71.3
	Week 6	Week 12	IL4	55	54
			IL10	78	67.5
			IL13	87	66.4
**Body weight**	Week 3	Week 15	--	31.4	30.6
	Week 6	Week 15		31.4	32.12
**Blood glucose**	Week 3	Week 12	--	160	158.05
	Week 6	Week 12		160	158.24

### Deriving mathematical scheme for evaluating biological formulation for obesity and diabetes

The model is ready to predict late time point body parameters, like body weight and blood glucose level, from early gene expression values obtained from new experiments. We could check the predictive ability of the models by comparing the predicted values with the experimentally obtained results. For comparison, the unknown experimental data need to be normalized with the reference data (the data used to build the model). For this normalization, we took the body parameters of the ND and HFHSD mice as the minimum and maximum values of the experimental range and converted them to the range of the reference experiment. Since this is a linear shift in scale and range, we used the Celsius-Fahrenheit conversion concept. Let the body weight of the ND mice of the reference experiment be *x*_*1*_ gram and HFHSD mice be *x*_*2*_ gram. Let the body weight of the ND mice of the new experiment be *y*_*1*_ gram and HFHSD be *y*_*2*_ gram. Since there is a linear relation between the ND of the model reference with the new experiment (*x*_*1*_, *y*_*1*_), and HFHSD of the model reference with the new experiment (*x*_*2*_, *y*_*2*_), so these two points will pass through a line. Let the equation of the line be *y = mx + c*, where *m* is the slope of the line and *c* is the intercept of the line. To get the equation we need to find the unknown parameter *m* and *c*. For this we substituted (*x*, *y*) from the equation of the line with (*x*_*1*_, *y*_*1*_) and (*x*_*2*_, *y*_*2*_) and got

(1) $$y_{1}=m\;x_{1}+c$$

(2) $$y_{2}=mx_{2}+c$$

Solving equation (1) and equation (2) we obtained *m* and *c* which we was used to convert the values of the new experiment to the referred experiment. These converted predicted values were then compared with the experimental observations.

Moreover, to make the model useful in terms of evaluating biological formulations for obesity and diabetes, we defined reference ranges for the body parameters distinguishing normal mice from obese or diabetic mice. We divided the mice into 3 groups depending on body parameter value. In our reference experiment, the body weight of the ND mice was 27.5 gram and the body weight of the HFHSD mice was 38 gram. So, the first group consisted of mice considered as normal body weight ranges between 27.5 gram to 30.125 gram (25% away from ND and 75% from HFHSD). Second group consisted of mice with body weight between 30.125 gram to 32.75 gram (50% from ND and HFHSD). The mice in this group showed sign of possible obesity. For body weight more than 32.75 gram, the mice were said to be obese and we put them in third group. Similarly we proposed ranges for the blood glucose level. In the reference experiment, ND mice had blood glucose level equal to 141 mg/dl and HFHSD was equal to 170 mg/dl. If the glucose level lies between 141 mg/dl and 148.25 mg/dl, we referred it as normal. Second group consisted of mice with slight increase in blood glucose level (148.25 mg/dl to 155.5 mg/dl). For blood glucose level more than 155.5 mg/dl, the mice were said to be diabetic and we put them in the third group. If the biological formulation works and keeps the body parameters within the normal range then we denote it by ‘+’. If the formulation partially works on the body parameters and the values are in the second group (described above), we denote it by ‘+/−’. Finally, in spite of exposing to the formulation, if the body parameter shows obese and or diabetic state then we denote it by ‘−’.

### Model predictions on the effectiveness of unknown formulations for obesity and diabetes

We performed a blind experiment where the goal was to predict outcomes of HFHSD mice treated with three different ethno-botanical formulations of unknown function. These three blind formulations, named as KAL-A, KAL-B, and KAL-C, were obtained from the manufacturer of KAL-1 and the data, in terms of the physiological parameters, were generated in a similar manner. Gene-expression data were generated from the skeletal muscle from different groups at weeks 3 and 6. We also obtained body parameters (body weight and blood glucose level) for later time point. The body weight and blood glucose of ND mice were 28 grams (week 15) and 138 mg/dl (week 12) respectively, and the body weight and blood glucose of HFHSD mice were 31 grams (week 15) and 158 mg/dl (week 12), respectively. Thus the conversion parameters (*m* and *c*) for the body weight and blood glucose level for this new experiment were (3.5, −70.5) and (1.45, −59.1), respectively. We used these two parameters to compare our prediction with the observed results. The model predictions for the new experiment (KAL-A, KAL-B and KAL-C) are given in Table 4. We observed that our predictions regarding the effectiveness of the biological formulations match with the experimental observations, except in case of border line predictions.

**Table 4:** Body weight (in grams) and blood glucose level (in mg/dl) of mice exposed with KAL-A, KAL-B and KAL-C formulations

**Formulation**	**Predicted body weight of the mice**	**Effectiveness (Predicted)**	**Experimentally observed body weight of the mice**	**Experimentally observed body weight of the mice (converted in the range to match the reference body weight range)**	**Effectiveness (Actual)**
**KAL A**	30.89	**+/−**	28.5	29.2	**+**
**KAL B**	32.02	**-**	30.8	37.3	**-**
**KAL C**	29.72	**+**	29	31	**+**
**Formulation**	**Predicted blood glucose level of the mice**	**Effectiveness (Predicted)**	**Experimentally observed blood glucose level of the mice**	**Experimentally observed blood glucose level of the mice (converted in the range to match the reference body weight range)**	**Effectiveness (Actual)**
**KAL A**	166.33	**-**	165	180.15	**-**
**KAL B**	166.17	**-**	165	180.15	**-**
**KAL C**	164	**-**	161	174.35	**-**

To check the robustness of the proposed models, we further tested them with ten more blind formulations (NF_1, NF_2, NF_3, NF_4, NF_5, NF_6, NF_7, NF_8, NF_9 and NF_10). The body weight and blood glucose of ND mice were 29 grams and 140 mg/dl respectively. The body weight and blood glucose of HFHSD mice were 34.5 grams and 169.5 mg/dl, respectively. Thus the conversion parameters (*m* and *c*) for the body weight and blood glucose level for this new experiment were (1.0972, −8.0273) and (1.017, −1.38) respectively. The model prediction for the new experiment with ten blind formulations is given in Tables 5 and 6. We observed that most of the predictions were correct except for some border line cases. Thus, the trained model showed a good first approximation on evaluating the effectiveness of the formulations against obesity and diabetes. Although with the failure in some border line cases, implied the need for further training of the model. Nevertheless, we have a model (trained and tested) that can be used as first-line screen to test the activities of formulations (including pharmacological compounds).

**Table 5:** Body weights (in grams) of mice fed with 10 unknown formulations

**Formulation**	**Predicted body weight of the mice**	**Effectiveness (predicted)**	**Experimentally observed body weight of the mice**	**Experimentally observed body weight of the mice (converted in the range to match the reference body weight range)**	**Effectiveness (Actual)**
**NF_1**	31.76	+/−	36.7	32.24	+/−
**NF_2**	32.89	-	38.88333	34.64	-
**NF_3**	33.1	-	37.63333	33.264	-
**NF_4**	31.75	+/−	39.3	35.0927	-
**NF_5**	31.48	+/−	41.43333	37.43	-
**NF_6**	31.21	+/−	41	36.96	-
**NF_7**	31.94	+/−	39.98333	35.84	-
**NF_8**	31.07	+/−	36.95	32.51	+/−
**NF_9**	30.57	+/−	36.68333	32.22	+/−
**NF_10**	31.24	+/−	34.86667	30.22	+/−

Second column has predicted values form the model and the fourth column is the observed values of the model. ‘+’ means the formulation works and ‘-’ means it does not work and ‘+/−’ means may or may not work.

**Table 6:** Blood glucose level (in mg/dl) of mice fed with 10 unknown formulations

**Formulation**	**Predicted blood glucose level from the model**	**Effectiveness (Predicted)**	**Experimentally observed blood glucose level**	**Experimentally observed blood glucose level of the mice (converted in the range to match the reference body weight range)**	**Effectiveness (Actual)**
**NF_1**	150.087	+/−	153.8333	155.068	+/−
**NF_2**	155.671	-	168	169.48	-
**NF_3**	161.86	-	159.5	160.83	-
**NF_4**	151.751	+/−	152.6667	153.88	+/−
**NF_5**	160.533	-	167.3333	168.8	-
**NF_6**	160.029	-	175.3333	176.93	-
**NF_7**	158.374	-	166.6667	168.12	-
**NF_8**	160.225	-	155.8333	157.1	-
**NF_9**	166.757	-	153.1667	154.4	+/−
**NF_10**	154.106	+/−	150.1667	151.34	+/−

Second column has predicted values form the model and the fourth column is the observed values of the model. ‘+’ means the formulation works and ‘-’ means it does not work and ‘+/−’ means may or may not work.

### Finding relation between biological processes associated with the signature genes and the body phenotype

The genes present in the proposed models (selected by the VIP score) could possibly regulate or influence the related physiological parameters. To find the functionality associated with these genes we looked for functional enrichment of the data. We analyzed the protein functional clusters using the online tool called PANTHER (Protein Analysis through Evolutionary Relationships; http://www.pantherdb.org) Classification System. PANTHER is a ontology tool where proteins are classified into families and subfamilies of shared function, which are further assigned to specific ontology terms in the two main categories- biological process and molecular function [16].

**Figure 4:**
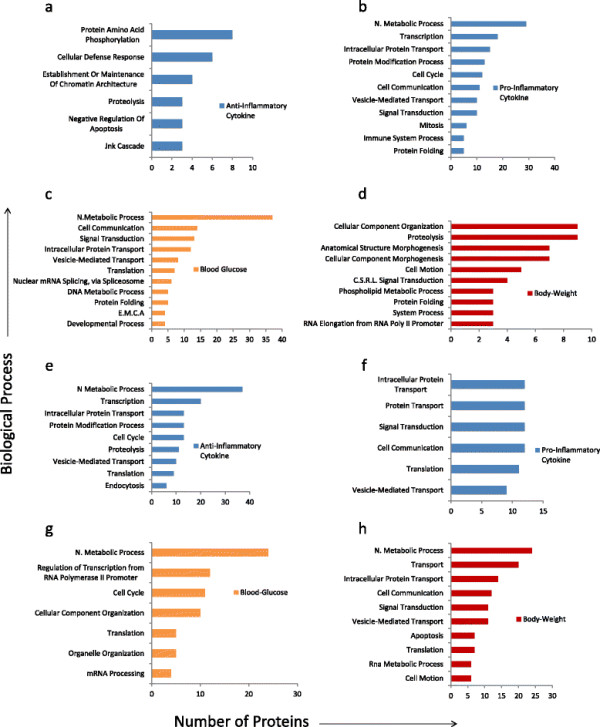
**The biological classification for the selected genes used in the models. (a)**--**(d)** The genes selected from the models for the adipose tissue (anti and pro-inflammatory cytokines, blood glucose and body weight). **(e)** -- **(h)** the genes selected from the models for the skeletal tissue (anti and pro-inflammatory cytokines, blood glucose and body weight). Here, the abbreviations means: N. Metabolic process- Nucleobase, Nucleoside, Nucleotide and Nucleic Acid Metabolic Process; C.S.R.L- Cell Surface Receptor Linked; E M C A- establishment or maintenance of chromatin architecture.

Here, we have eight set of genes (each set for each model) for skeletal and adipose SA tissue. Each of these sets are related (through model) with one of the parameter- (1) body weight, (2) blood glucose, (3) anti-inflammatory cytokines and (4) pro-inflammatory cytokines. In each group, we identified the key processes that were associated with these proteins, using the Panther HMM algorithm based on homology and trained on known proteins, see Figure 4. To identify these key processes, we first selected the processes based on significant p values (<1E-01) and the resulting list was further screened by selecting only those processes that involved more than 2 proteins. The list of biological processes obtained for each set of signature gene is given in Additional file 4.

We observed that there were some processes significantly (with p values <1E-01) present in both tissues (adipose and skeletal) and influence the same physiological parameter. For example, cell communication (with p-value 4.88E-03 in adipose and 1.92E-02 in skeletal), signal transduction (with p-value 4.27E-03 in adipose and 3.35E-02 in skeletal) and vesicle mediated transport (with p-value 2.22E-02 in adipose SA and 3.91E-02 in skeletal) were the key processes involve in both tissues and influence pro-inflammatory cytokine. So, there are lot of initial cell signalling events occurred that influence later time points pro-inflammatory cytokine profiles and this happens in both tissues. We also observed processes like proteolysis and cell motion that were significantly present in both the tissues. Former related to anti-inflammatory cytokines and later influences body weight. For blood glucose we observed translation and nucleobase, nucleoside, nucleotide and nucleic acid metabolic process significantly present in both the tissues.

There was no process commonly involved in all physiological changes for both the tissues, but there were processes uniquely present in each tissue and were involved in a particular physiological change. For example, genes involved in phospholipid metabolic process (9.38E-02) were present in adipose tissue influencing the body weight. This process was involved in the formation of lipid bilayers. Two more processes significantly present in adipose tissue were cellular defence response (9.58E-03) and regulation of apoptosis (9.18E-02) and they influenced anti-inflammatory cytokine. We also observed immune response (5.83E-02) in adipose tissue which influences the pro-inflammatory cytokine profiles. In skeletal tissues the key process includes transport (3.21E-02) like intra cellular protein transport and apoptosis (8.07E-02).

### Extracting biological network from the signature genes and identification of hub proteins

We obtained relations between different biological processes and body parameters like body weights, blood glucose levels and cytokine profiles. These processes were obtained from the genes present in the early time point and the body parameters were taken from later time point. This kind of linkage can trace disease development and progression, but for that we need to understand how the perturbations in a protein affect other proteins and how these changes are reflected on the body parameter. For this, we need to construct the protein-protein interaction (PPI) functional network. We used STRING (Search Tool for the retrieval of Interacting genes/ proteins) database, which detect physical and functional associations of the proteins and shows connection between two proteins if they are co-regulated, co-expressed or present in the same protein complex. We downloaded protein-protein interaction from STRING data set and used only those interactions that were obtained directly from experiment. (http://string-db.org/).

**Figure 5:**
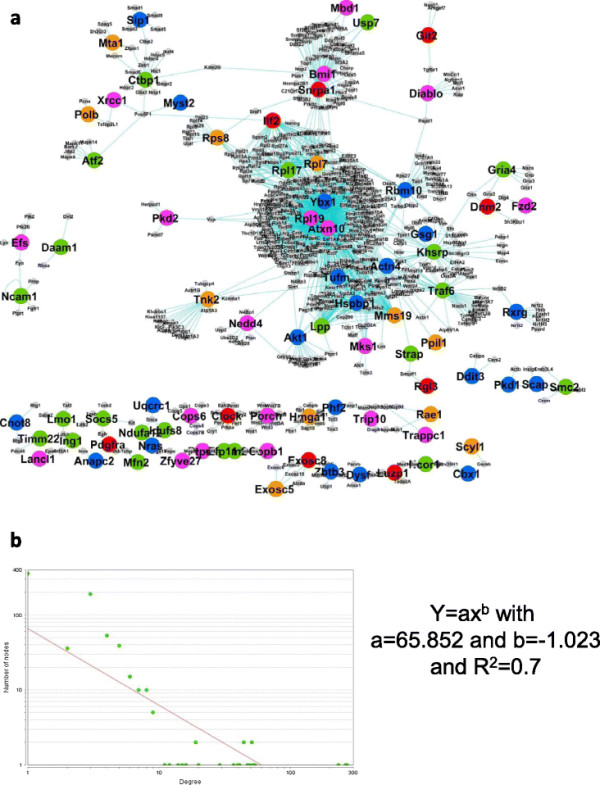
**Functional PPI network for skeletal tissue. (a)** The PPI network with different coloured nodes (showing their influence over different physiological parameters) connected through their interactors extracted from STRING data base. Nodes uniquely related to a particular physiological parameter are: Red nodes—related to body weight, green nodes—related to blood glucose, blue nodes—related to anti- inflammatory cytokines and reddish yellow nodes— related to pro-inflammatory cytokines. Magenta coloured nodes are for genes influencing more than one physiological parameter. **(b)** The power law distribution of the network showing its “scale-free” property.

**Figure 6:**
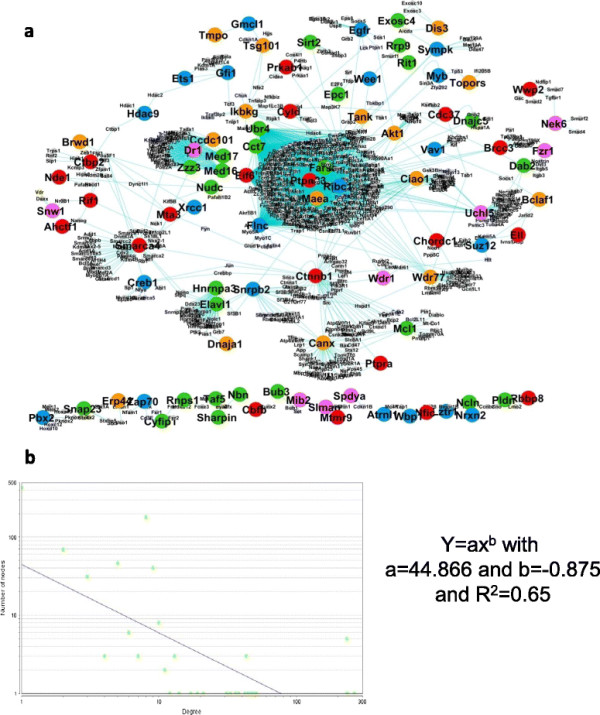
**Functional PPI network for adipose SA tissue. (a)** The PPI network with different coloured nodes (showing their influence over different physiological parameters) connected through their interactors extracted from STRING data base. Colour codes are same as in Figure 5. **(b)** The power law distribution of the network showing its “scale-free” property.

We built two PPI networks for the two tissues- skeletal and adipose, by combining the signature genes from all the models (for each tissue). The nodes (present in our list) were represented with different colours and were connected through their interactors extracted from STRING database, see Figures 5(a) and 6(a). In our constructed network we go for different statistical analysis. The first one is the degree-node distribution. The degree distribution is an important statistical property to measure the global structure of large networks [17]. The degree distribution, *P(k)*, describes the probability that a node has degree *k*. A scale-free networks (important property relevant to PPI network [14]) have a power-law degree distribution *P(k)* ∼ c*k*^−*γ*^ , where *γ* is a positive number [17]–[19]. Our network also showed power law distribution (see Figures 5(b)-6(b)) and hence it possesses “scale free” property.

In both the networks we obtained different enriched sub-networks based on the functional classification with low p-value (<10^−1^) obtained using PANTHER. To test the statistical significance of the sub-networks in terms of connectivity, we calculated the parameters like network centralization, network density and network heterogeneity for each of the proposed sub networks. These parameters are related with node connectivity, for example, centralization is an index of the connectivity distribution and network heterogeneity are based on the variance of the connectivity [20]. Moreover in each of these sub-networks (or functional modules) we identified some hub proteins. Here we focused on intra-modular hub proteins which has high clustering coefficient [21].

### Functional modules in skeletal tissue

**Figure 7:**
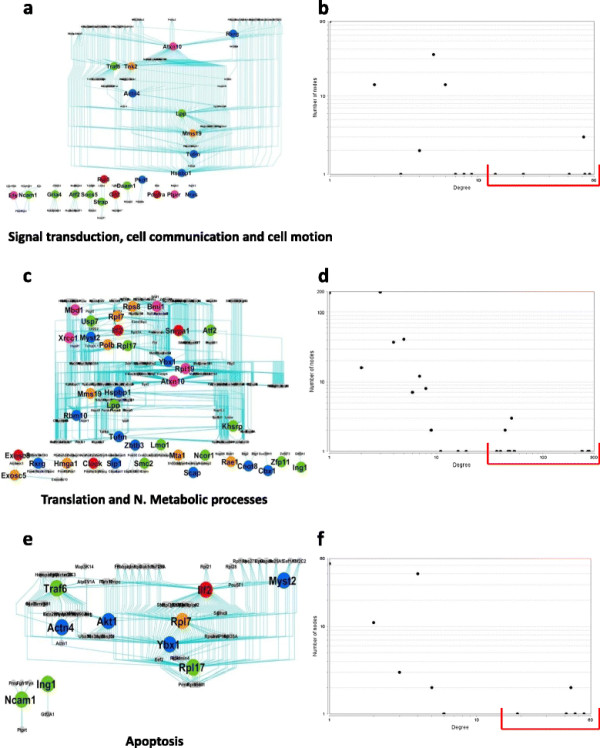
**Functionally defined modules extracted from the skeletal network given in Figure**5**(a). (a)**-**(b)** Signal transduction, cell communication and cell motion; **(c)**-**(d)** Translation and N. Metabolic processes; and **(e)**-**(f)** Apoptosis. **(a)**, **(c)** and **(e)** are the sub-networks and **(b)**, **(d)** and **(f)** are the corresponding node-degree distribution, using which we obtained the hub proteins.

There were mainly three functionally sub-networks observed in this network. The statistics for each sub-network is given in Table 7. First one was “Signal transduction, cell communication and cell motion” which contains proteins that play a central role in signalling pathway (see Figure 7(a)). From the node-degree distribution, we obtained the average neighbourhood for each node and took eight nodes (top 5%, see Figure 7(b)) as hub-nodes, see Table 8. The second sub-network was “Translation and nucleobase, nucleoside, nucleotide and nucleic acid metabolic processes” containing proteins that play a central role in metabolic pathway (see Figure 7(c)). From the node-degree distribution, we obtained ten nodes (top 2%, see Figure 7(d)) as hub-nodes, see Table 8. The third sub-network plays a central role in apoptosis (see Figure 7(e)) and we obtained six nodes (top 5%, see Figure 7(f)) as hub-nodes, see Table 8.

**Table 7:** **The parameters related with node connectivity for the sub-networks extracted from the skeletal network given in Figure**5**(a)**

**Biological sub-network**	**Number of nodes**	**Clustering coefficient**	**Network centralization**	**Avg. number of neighbours**	**Network density**	**Network heterogeneity**
Signal transduction, cell communication and cell motion	166	0.28	0.316	4.46	0.062	2.083
Translation and N. Metabolic processes	526	0.516	0.507	5.038	0.05	3.967
Apoptosis	115	0.39	0.443	4.4	0.04	2.081

**Table 8:** **The list of hub proteins obtained in different modules in the skeletal network given in Figure**5**(a)**

**Biological sub-network**	**Number of hub nodes**	**Name of the proteins**	**Number of nodes connected with**
Signal transduction, cell communication and cell motion	8	Atxn10	56
		Lpp	52
		Mms19	19
		Tufm	51
		Hspbp1	51
		Traf6	41
		Tnk2	20
		Actn4	13
Translation and N. metabolic processes	10	Ybx1	270
		Atxn10	263
		Rpl19	231
		Mms19	54
		Ilf2	47
		Snrpa1	49
		Rpl17	44
		Rp17	44
		Lpp	51
		Hspbp1	51
Apoptosis	6	Traf6	41
		IIf2 d	47
		Rp17	44
		Ybx1	54
		Rpl17	44
		Akt1	19

### Functional modules in adipose tissue

**Figure 8:**
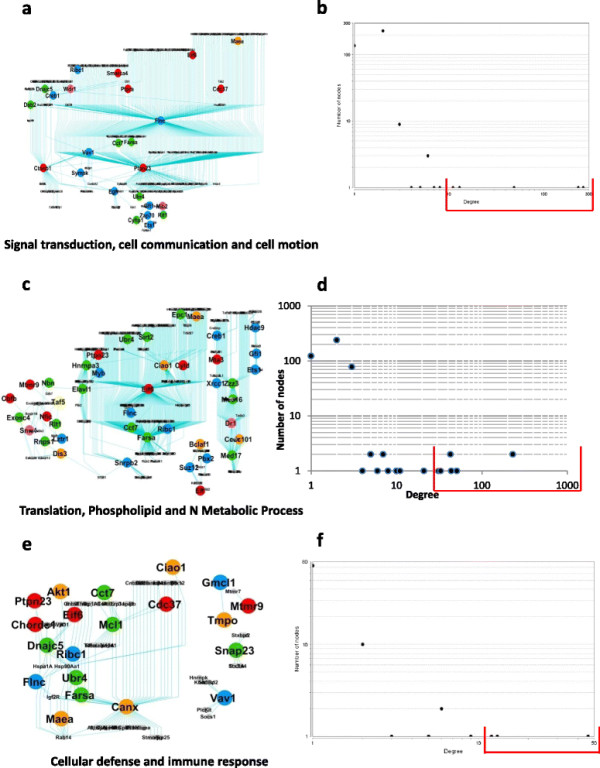
**Functionally defined modules extracted from the adipose SA network given in Figure**6**(a). (a)**-**(b)** Signal transduction, cell communication and cell motion; **(c)**-**(d)** Translation, N. Metabolic processes and Phospho-lipid Metabolic Process; and **(e)**-**(f)** Immune response. **(a)**, **(c)** and **(e)** are the sub-networks and **(b)**, **(d)** and **(f)** are the corresponding node-degree distribution, using which we obtained the hub proteins.

Here also three functionally sub-networks were observed and the statistics for each sub-network is given in Table 9. First one was “Signal transduction, cell communication and cell motion” which contained proteins from signalling pathway (see Figure 8(a)). From the node-degree distribution, we obtained five nodes (2%, see Figure 8(b)) as hub-nodes, see Table 10. The second sub-network was “Translation, N Metabolic Process, Phospholipid metabolic Process” that play important part in different metabolic process (see Figure 8(c)). Here we obtained eight nodes (top 2%, Figure 8(d)) as hub-nodes, see Table 10. The third sub-network consisted of proteins that were part of immune response pathway (see Figure 8(e)). We obtained three nodes (top 3%, see Figure 8(f)) as hub-nodes, see Table 10.

**Table 9:** **The parameters related with node connectivity for the sub-networks extracted from the adipose SA network given in Figure**6**(a)**

**Biological sub-network**	**Number of nodes**	**Clustering coefficient**	**Network centralization**	**Avg. Number of neighbours**	**Network density**	**Network heterogeneity**
Cell communication, signal transduction, cell motion	386	0.583	0.676	3.176	0.008	5.608
Translation, N. Metabolic process, Phospho-lipid Metabolic process	459	0.613	0.499	3.542	0.008	4.448
Immune response	90	0.023	0.504	2.111	0.024	2.398

**Table 10:** **The list of hub proteins obtained in different modules in adipose SA network given in Figure**6**(a)**

**Biological sub-network**	**Number of hub nodes**	**Name of the proteins**	**Number of nodes connected with**
Cell communication, signal transduction, cell motion	5	Flnc	262
		Ptpn23	232
		Ctnnb1	47
		Egfr	13
		Creb1	11
Translation, N. Metabolic process, Phospho-lipid metabolic process	8	Eif6	231
		Farsa	231
		Ciao1	51
		Zzz3	44
		Dr1	43
		Med17	43
		Elavl1	33
		Hnrnpa3	31
Immune response	3	Canx	46
		Hsp90Aa1	13
		Mcl1	12

## Discussion

Present study was devoted in finding gene signatures for obesity and diabetes systemic inflammation. Using the partial least square discriminant analysis (PLS-DA) method, we identified early gene/molecular signatures that predicted future values of physiological parameters like body weight, blood glucose levels and cytokine profiles. The models worked better for Skeletal & Adipose SA tissues in predicting body weight and blood glucose (two important physiological parameters for obesity and diabetes). To test the effectiveness of the biological formulations against obesity and diabetes, we set cut-off values for the body weights and blood glucose levels. It was observed that the models, with these cut offs, work well in evaluating the formulations, except in some border cases for which the model needed further training. Thus, we have models that predict future body weight and blood glucose level from early gene expression values, which could be a good starting platform for screening experiment.

We also studied the functionality of the obtained signature genes for each model. We observed that some of the biological processes were common in both the tissues and influenced more than one physiological parameter. There were biological processes uniquely present in a tissue and were associated with a particular physiological parameter. After identifying the significant biological processes (p-value < 1E-01) for both skeletal and adipose SA, we used them to build functional modules for both the tissues (using STRING database). Studying those modules carefully we obtained hub proteins with some of them has the potential to be an early marker for obesity and diabetes.

For skeletal tissues we obtained 15 unique hub proteins from different modules, influencing different future physiological parameters and for adipose tissue we obtained 16 unique hub proteins, see Additional file 5 (proteins with their functional modules and the physiological parameters).

We observed heat shock proteins Hspbp1 in skeletal tissue (signaling and metabolic module) influencing future anti-inflammatory cytokine profiles. Heat shock proteins (HSPs) function at the cellular level to protect cells against many chronically and acutely stressful conditions [22]. Thus response to early stress due to HFHSD was observed in the mice. Another key protein identified in apoptosis pathway and influence future blood glucose level was Akt1. We know that Akt1 phosphorylation is essential for insulin signaling in skeletal muscle [23]. Our study identified Akt1 as a key signature gene in skeletal tissue for predicting future blood glucose level. Another key signature gene in skeletal tissue was Ybx1 which predicts future anti-inflammatory cytokine profiles. This is an interesting result because Ybx1 is an important molecule associated with inflammatory stress [24] and here its early expression in the skeletal tissue was used to predict the future outcome in terms of inflammation. We have also identified some key ribosomal proteins (Rpl17, Rpl19, and Rp17) as signature molecules for predicting future inflammatory stress.

In adipose SA we found key proteins from signaling pathway, metabolic pathway and immune response. Immune response and metabolic regulation are highly integrated function and any kind of dysfunction may lead to a cluster of chronic metabolic disorders, particularly obesity, type-II diabetes and cardiovascular disease [25]. In immune response module we found a highly connected molecule Calnexin which is an important molecule found in obesity [26]. We identified a key signature protein Flnc (highly connected in the signaling module) associated with future inflammation stress. This protein is known to associate with obesity and diabetes [27]. Ctnnb1 is another important protein identified in the signaling module, known as an important players in Wnt and p53 signalling pathways, which would provide a putative link between Type-II diabetes and certain types of cancer [28]. This identified key signature is observed to influence the future body weight.

## Conclusion

We proposed sets of gene/molecular signatures for predicting different physiological parameters associated with obesity and diabetes. This could be used as a first step in evaluating effectiveness of the formulations against obesity and diabetes. Our analysis revealed that some of the identified key signature genes have the potential to become early biomarker for obesity and diabetes, admittedly however, it necessitates further validation.

## Competing interests

The authors declare that they have no competing interests.

## Authors’ contributions

NS performed the PLSR analysis and network analysis. SS, PT and KT isolated tissues and extracted RNA for micro-array. SKN performed the initial data analysis and PLSR analysis. DK designed the PLSR analysis. KVSR conceived and managed the overall project. SC designed the initial data analysis and PLSR analysis, performed the network analysis, interpreted the results and wrote the manuscript. All authors read and approved the final manuscript.

## Additional files

## Supplementary Material

Additional file 1: Master Gene List. Click here for file

Additional file 2: Appendix for variable importance. Click here for file

Additional file 3: Gene list as input in the models. Click here for file

Additional file 4: Biological process/pathways. Click here for file

Additional file 5: Key proteins and their functional modules. Click here for file

## References

[B1] http://www.who.int/nmh/publications/ncd_report2010/en/Alwan A: **The World Health Report.**.

[B2] Hill JO Peters JC Catenacci VA Wyatt HR International strategies to address obesity Obes Rev 2008 9 Suppl 1 41 47 10.1111/j.1467-789X.2007.00437.x 18307698

[B3] Haslam DW James WPT Obesity Lancet 2005 366 9492 1197 1209 10.1016/S0140-6736(05)67483-1 16198769

[B4] Kopelman PG Obesity as a medical problem Nature 2000 404 6778 635 643 10766250 10.1038/35007508

[B5] Shao W Yu Z Chiang Y Yang Y Chai T Foltz W Lu H Fantus IG Jin T Curcumin prevents high fat diet induced insulin resistance and obesity via attenuating lipogenesis in liver and inflammatory pathway in adipocytes PLoS One 2012 7 1 e28784 10.1371/journal.pone.0028784 22253696 PMC3253779

[B6] Tikoo K Misra S Rao KV Tripathi P Sharma S Immunomodulatory Role of an Ayurvedic Formulation on Imbalanced Immunometabolics during Inflammatory Responses of Obesity and Prediabetic Disease Evid base Compl Alternative Med 2013 2013 795072 10.1155/2013/795072 PMC3835817 24302970

[B7] Perez-Enciso M Tenenhaus M Prediction of clinical outcome with microarray data: a partial least squares discriminant analysis (PLS-DA) approach Hum Genet 2003 112 5–6 581 592 12607117 10.1007/s00439-003-0921-9

[B8] Lin S Thomas TC Storlien LH Huang XF Development of high fat diet-induced obesity and leptin resistance in C57Bl/6 J mice Int J Obes Relat Metab Disord 2000 24 5 639 646 10.1038/sj.ijo.0801209 10849588

[B9] Petro AE Cotter J Cooper DA Peters JC Surwit SJ Surwit RS Fat, carbohydrate, and calories in the development of diabetes and obesity in the C57BL/6 J mouse Metabolism 2004 53 4 454 457 10.1016/j.metabol.2003.11.018 15045691

[B10] Surwit RS Feinglos MN Rodin J Sutherland A Petro AE Opara EC Kuhn CM Rebuffe-Scrive M Differential effects of fat and sucrose on the development of obesity and diabetes in C57BL/6 J and A/J mice Metabolism 1995 44 5 645 651 10.1016/0026-0495(95)90123-X 7752914

[B11] Staehr P Hother-Nielsen O Beck-Nielsen H The role of the liver in type 2 diabetes Rev Endocr Metab Disord 2004 5 2 105 110 10.1023/B:REMD.0000021431.90494.0c 15041785

[B12] Juge-Aubry CE Henrichot E Meier CA Adipose tissue: a regulator of inflammation Best Pract Res Clin Endocrinol Metab 2005 19 4 547 566 10.1016/j.beem.2005.07.009 16311216

[B13] Lin Y Sun Z Current views on type 2 diabetes J Endocrinol 2010 204 1 1 11 10.1677/JOE-09-0260 19770178 PMC2814170

[B14] Barabasi AL Albert R Emergence of scaling in random networks Science 1999 286 5439 509 512 10.1126/science.286.5439.509 10521342

[B15] Korhonen O Matero S Poso A Ketolainen J Partial least square projections to latent structures analysis (PLS) in evaluating and predicting drug release from starch acetate matrix tablets J Pharm Sci 2005 94 12 2716 2730 10.1002/jps.20485 16258997

[B16] Mi H Thomas P PANTHER pathway: an ontology-based pathway database coupled with data analysis tools Methods Mol Biol 2009 563 123 140 10.1007/978-1-60761-175-2_7 19597783 PMC6608593

[B17] Przulj N Corneil DG Jurisica I Modeling interactome: scale-free or geometric? Bioinformatics 2004 20 18 3508 3515 10.1093/bioinformatics/bth436 15284103

[B18] Barabasi A-L Oltvai ZN Network biology: understanding the cell’s functional organization Nat Rev Genet 2004 5 2 101 113 10.1038/nrg1272 14735121

[B19] Yook SH Oltvai ZN Barabasi AL Functional and topological characterization of protein interaction networks Proteomics 2004 4 4 928 942 10.1002/pmic.200300636 15048975

[B20] Dong J Horvath S Understanding network concepts in modules BMC Syst Biol 2007 1 24 10.1186/1752-0509-1-24 17547772 PMC3238286

[B21] Liang H Li WH MicroRNA regulation of human protein protein interaction network RNA 2007 13 9 1402 1408 10.1261/rna.634607 17652130 PMC1950750

[B22] Chung J Nguyen AK Henstridge DC Holmes AG Chan MH Mesa JL Lancaster GI Southgate RJ Bruce CR Duffy SJ Horvath I Mestril R Watt MJ Hooper PL Kingwell BA Vigh L Hevener A Febbraio MA HSP72 protects against obesity-induced insulin resistance Proc Natl Acad Sci U S A 2008 105 5 1739 1744 10.1073/pnas.0705799105 18223156 PMC2234214

[B23] Lee JH Ragolia L AKT phosphorylation is essential for insulin-induced relaxation of rat vascular smooth muscle cells Am J Physiol Cell Physiol 2006 291 6 C1355 C1365 10.1152/ajpcell.00125.2006 16855220 PMC1636679

[B24] Shelton RC Claiborne J Sidoryk-Wegrzynowicz M Reddy R Aschner M Lewis DA Mirnics K Altered expression of genes involved in inflammation and apoptosis in frontal cortex in major depression Mol Psychiatry 2011 16 7 751 762 10.1038/mp.2010.52 20479761 PMC2928407

[B25] Hotamisligil GS Inflammation and metabolic disorders Nature 2006 444 7121 860 867 10.1038/nature05485 17167474

[B26] van Noort V Snel B Huynen MA The yeast coexpression network has a small-world, scale-free architecture and can be explained by a simple model EMBO Rep 2004 5 3 280 284 10.1038/sj.embor.7400090 14968131 PMC1299002

[B27] Kaput J Klein KG Reyes EJ Kibbe WA Cooney CA Jovanovic B Visek WJ Wolff GL Identification of genes contributing to the obese yellow Avy phenotype: caloric restriction, genotype, diet x genotype interactions Physiol Genomics 2004 18 3 316 324 10.1152/physiolgenomics.00065.2003 15306695

[B28] Jesmin J Rashid MS Jamil H Hontecillas R Bassaganya-Riera J Gene regulatory network reveals oxidative stress as the underlying molecular mechanism of type 2 diabetes and hypertension BMC Med Genomics 2010 3 1 45 10.1186/1755-8794-3-45 20942928 PMC2965702

