# Supplementary material for: Molecular signatures for obesity and associated disorders identified through partial least square regression models
Source: BMC Syst Biol. 2014 Aug 30;8:104. doi: 10.1186/s12918-014-0104-4 (PMC4363939; doi:10.1186/s12918-014-0104-4)
Supplement: Additional file 2: — Appendix for variable importance. [file s12918-014-0104-4-S2.pdf]

Appendix

Variable importance (VIP)

VIP is the sum over all model dimensions of the contribution VIN (variable influence). For a given PLS dimension, a, $(VIN)_{ak2}$ is equal to the squared PLS weight $(W_{ak})^2$ of that term, multiplied by the explained SS of that PLS dimension. The accumulated (overall PLS dimensions) value,

$$VIP_{ak}^2 = \Sigma(VIN)_{ak}^2$$

Where the summation is made over a= 1 to A.

This value is then divided by the total explained SS by the PLS model and multiplied by the number of terms in the model. The final VIP is square root of that number. The formula can also be expressed as:

$VIP_{AK} = sqrt\ (\sum (w_{ak}^2 * (SSY_{a-1} - SSY_k)) * (K / (SSY_a - SSY_A)))$

The sum of squares of all VIPs is equal to the number of terms in the model hence the average VIP is equal to 1. One can compare the VIP of one term to other. Terms with large VIP, larger than 1, are the most relevant for explaining Y.

To find optimal cut off value

We begin with a cut off value of 1 and ran the model with the selected genes. Then we gradually increase the cut off value of the VIP (and of course decreasing the number of gene selected) and ran the model. The procedure continues until we reach the number below which the model was not working or giving good prediction. This optimal cut off value is then used for selecting the genes for the final model.
