# Supplementary material for: Molecular signatures for obesity and associated disorders identified through partial least square regression models
Source: BMC Syst Biol. 2014 Aug 30;8:104. doi: 10.1186/s12918-014-0104-4 (PMC4363939; doi:10.1186/s12918-014-0104-4)
Supplement: Additional file 5: — Key proteins and their functional modules. [file s12918-014-0104-4-S5.pdf]

Skeletal tissue

key proteins	Functional modules	physiological parameters
Atxn10	Signaling pathway, Metabolic pathway	body weight, anti-inflammatory cytokines
Lpp	Signaling pathway, Metabolic pathway	blood glucose
Mms19	Signaling pathway, Metabolic pathway	pro-inflammatory cytokine
Hspbp1	Signaling pathway, Metabolic pathway	anti-inflammatory cytokines
Traf6	Signaling pathway, Apoptosis	blood glucose
Ybx1	Metabolic pathway, Apoptosis	anti-inflammatory cytokines
Ilf2	Metabolic pathway, Apoptosis	body weight
Rpl17	Metabolic pathway, Apoptosis	blood glucose
Rp17	Metabolic pathway, Apoptosis	pro-inflammatory cytokine
Tufm	Signaling pathway	anti-inflammatory cytokines
Tnk2	Signaling pathway	pro-inflammatory cytokine
Actn4	Signaling pathway	anti-inflammatory cytokines
Rpl19	Metabolic pathway	body weight, pro-inflammatory cytokines
Snrpa1	Metabolic pathway	body weight
Akt1	Apoptosis	blood glucose

Adipose SA tissue

key proteins	Functional modules	physiological parameters
Flnc	Signaling pathway	anti-inflammatory cytokines
Ptpn23	Signaling pathway	body weight
Ctnnb1	Signaling pathway	body weight
Egfr	Signaling pathway	anti-inflammatory cytokines
Creb1	Signaling pathway	anti-inflammatory cytokines
Eif6	Metabolic pathway	body weight
Farsa	Metabolic pathway	blood glucose
Ciao1	Metabolic pathway	pro-inflammatory cytokines
Zzz3	Metabolic pathway	blood glucose
dr1	Metabolic pathway	blood glucose, pro-inflammatory cytokines
Med17	Metabolic pathway	blood glucose
Elavl1	Metabolic pathway	blood glucose
Hnrnpa3	Metabolic pathway	blood glucose
Canx	Immune response	pro-inflammatory cytokines
Hsp90Aa1	Immune response	Not present in our list of signature genes
mcl1	Immune response	blood glucose, anti-inflammatory cytokines
